# ERCP in Evaluating The Mode of Therapy in Pancreatic Pseudocyst

**DOI:** 10.1155/1988/47060

**Published:** 1988

**Authors:** I. Nordback, O. Auvinen, I. Airo, J. Isolauri, O. Teerenhovi

**Affiliations:** 1 Department of Surgery University Central Hospital Tampere Finland; 2 Department of Surgery Institute of Clinical Sciences University of Tampere Box 607 Tampere 33101 Finland

## Abstract

Twenty patients with ultrasonographic or computed tomographic diagnosis of pancreatic pseudocyst were referred for endoscopic retrograde cholangiopancreatography (ERCP). Two of these were found at laparotomy not to have pseudocysts and were excluded. Pancreatography was successful in 15 out of 18 cases (83%) and cholangiography in 12 out of 18 cases (67%). Three types of pseudocysts were noticed according to the communication of the pseudocyst to the main pancreatic duct and the presence of pancreatic duct stensosis. Successful treatment included two spontaneous resolutions, two internal drainages and three left pancreatic resections. In the eight percutaneous external drainages four recurrences (50%) occurred, one after closure of temporary pancreatocutaneous fistula. All the recurrences occurred in Type III pseudocysts with communication of the pseudocysts to stenotic main pancreatic duct. In these cases internal drainage would have been the preferable treatment method. We believe that by ERCP one can identify pseudocysts not suitable for external drainage.

## INTRODUCTION

Pancreatic pseudocysts are classified as acute or chronic depending on whether they developed immediately after acute pancreatitis[1]. The time interval between the onset of acute pancreatitis and discovery of pseudocysts has been 1–6 weeks, while in patients with chronic symptomatic pseudocysts no episode of acute pancreatitis can be found. Most pseudocysts are acute (40–75%) with a spontaneous resolution frequency up to 40%[2], whereas 25–60% are chronic pseudocysts with a minimal (3%) spontaneous resolution rate[3]. Because of the considerable complication rate, active management has been recommended for pseudocysts over 4 cm in diameter[4].

Endoscopic retrograde cholangiopancreatography (ERCP) has been considered a valuable investigation method in planning the therapy of pseudocysts. The advantage of ERCP is the demonstration of the exact location and possible multiplicity of pseudocysts, and ductal anatomy and pathology in relation to the pseudocyst, as well as simultaneous biliary abnormalities[5–8]. It has been claimed that the pre-operative ERCP may modify tactics in 50% of patients[7]. Thus, better therapy results may be expected. Hence, we have here analyzed our experience of ERCP in pancreatic pseudocysts.

## METHODS

Between February 1984 and June 1987 ERCP was performed on 20 patients with suspected pseudocyst of the pancreas. At laparotomy one of these was found to be a mucinous cystadenoma of the pancreas and another pancreatic carcinoma and splenic cyst. Both cases were excluded. Three of the remaining ERCPs failed, twice due to duodenal compression caused by the pseudocyst and once due to technical failure in cannulation. Thus, the study material comprises 15 patients.

In all cases abdominal ultrasonography was performed. By this means pseudocyst was revealed in 10 cases, abdominal CT gave the diagnosis in four further cases, and in one case pseudocyst was not diagnosed until ERCP. The size of the pseudocyst varied from 2 to 15 cm (mean 7 cm; in four cases below 4 cm).

The ERCPs were performed by an Olympus JF IT 10 side-viewing duodenoscope. The patients were lying on their left in a prone position. The pancreatic duct was cannulated selectively in all cases. Common bile-duct cannulation was attempted but failed in three cases. A single dose of netilmycin (150 mg) and more recently (last seven cases) ceftazidime (1 g) was given intravenously immediately prior to ERCP. In cases with pseudocyst filling the drug was continued for three days. Diazepam (10 mg intravenously) was used for sedation, and glucagon (0.3 mg intravenously) or butylscopolamine (20 mg intravenously) for inhibiting duodenal peristalsis. The contrast medium was injected gently through sterile catheters under fluoroscopic control. The injection was discontinued when secondary side branches of the pancreatic duct or pseudocyst started to fill.

The mean time interval between ERCP and pseudocyst therapy was 13 days. The therapeutic approach was undertaken within 48 h in six cases. Table 1 shows data of the patients and pseudocysts.

The relation between ductal anatomy and pseudocyst was classified (Figure 1), modifying that presented by Sugava and Walt[5]. In type I the main pancreatic duct is imaged up to the end without much stenosis. The pseudocyst may (Type IA) or may not (IB) be filled, but is further away from the main pancreatic duct. In Type II there is again no main duct stenosis and pseudocyst opens to the duct. In Type III there is

**Table 1** Patients treated for pancreatic pseudocysts with preceding ERCP.

| | |
|---|---|
| Number of cases | 15 |
| Mean age (range) | 50 years (34–80) |
| Sex | |
| Men | 11 (73%) |
| | 4 (27%) |
| Pseudocysts | |
| Acute | 3 (20%) |
| Chronic | 12 (80%) |
| Aetiology | |
| Alcohol | 9 (60%) |
| Biliary | 4 (27%) |
| Other or unknown | 2 (13%) |
| Mean follow-up (range) | 8 months (2 months – 3 years) |

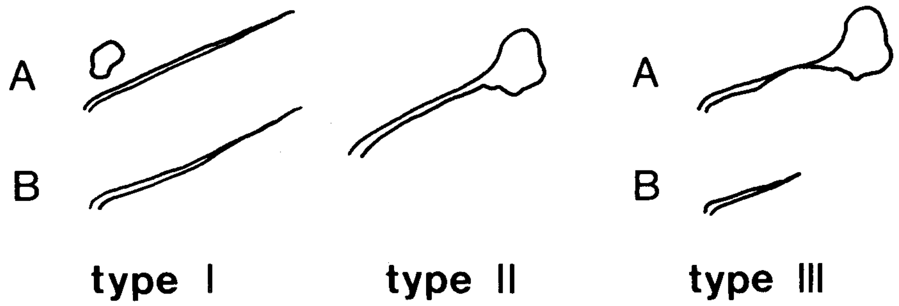

**Figure 1** Classification of pseudocysts according to the communication of the pseudocysts to the main pancreatic duct (Types II and IIIA, possibly Type IIIB) and the presence of stenosis in the main duct (Type III).

stenosis of the main pancreatic duct. There may again be filling of the pseudocyst behind the stenosis (IIIA), or not (IIIB). Pancreatograms of various types are presented in Figures 2–6.

Possible ERCP complications were investigated. The method of treatment and its complications were recorded. The resolution and possible recurrence of the pseudocyst were checked by ultrasonographic follow-up.

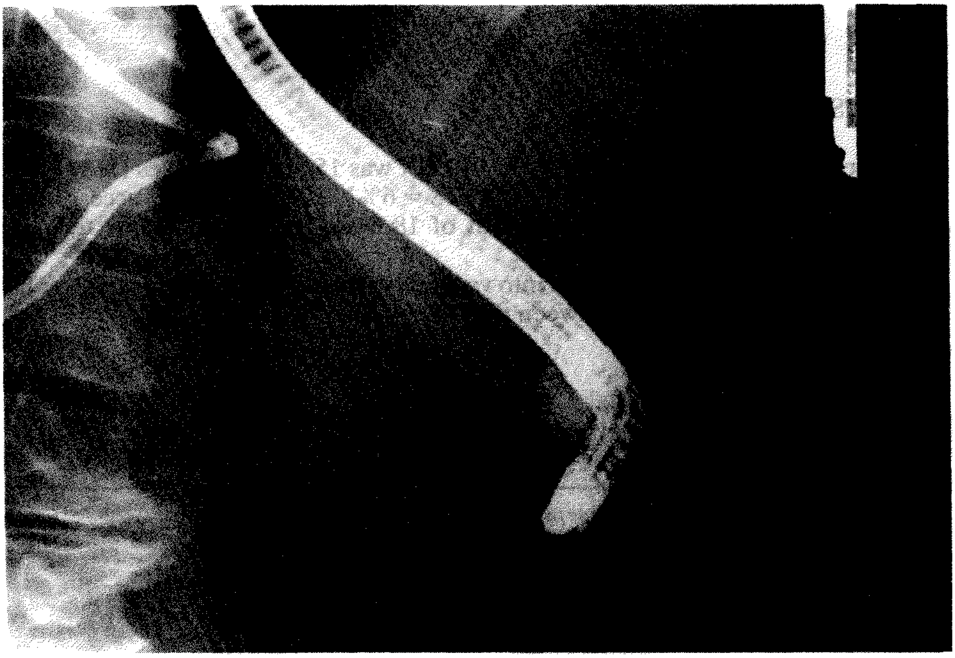

**Figure 2** Pancreatography of Type IA.

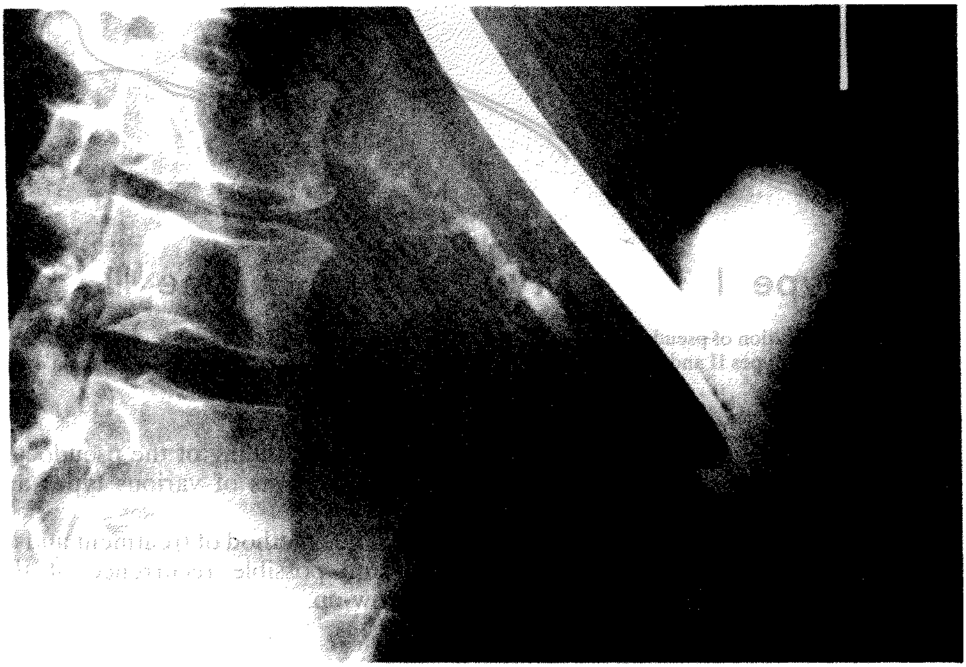

**Figure 3** Pancreatography of Type IB with severe chronic pancreatitis.

## RESULTS

The success rate in access of the pancreatic duct was 15 out of 18 cases (83%), with only one technical failure in cannulation (1/18 = 6%). The respective success rate in selective bil-duct cannulation was 12 out of 18 cases (67%), with four technical failures (22%).

The pseudocysts were distributed into various types as shown in Table 2. Over half (8/15) had main duct stenosis between the pseudocyst and papillary orifice.

**Table 2** Distribution of patients with pancreatic pseudocysts according to our classification presented in Figure 1.

| | | *Acute pseudocysts* | *Chronic pseudocysts* |
|---|---|---|---|
| Type I | A | 1 | |
| | B | | 2 |
| Type II | | 2 | 2 |
| Type III | A | | 4 |
| | B | | 4 |

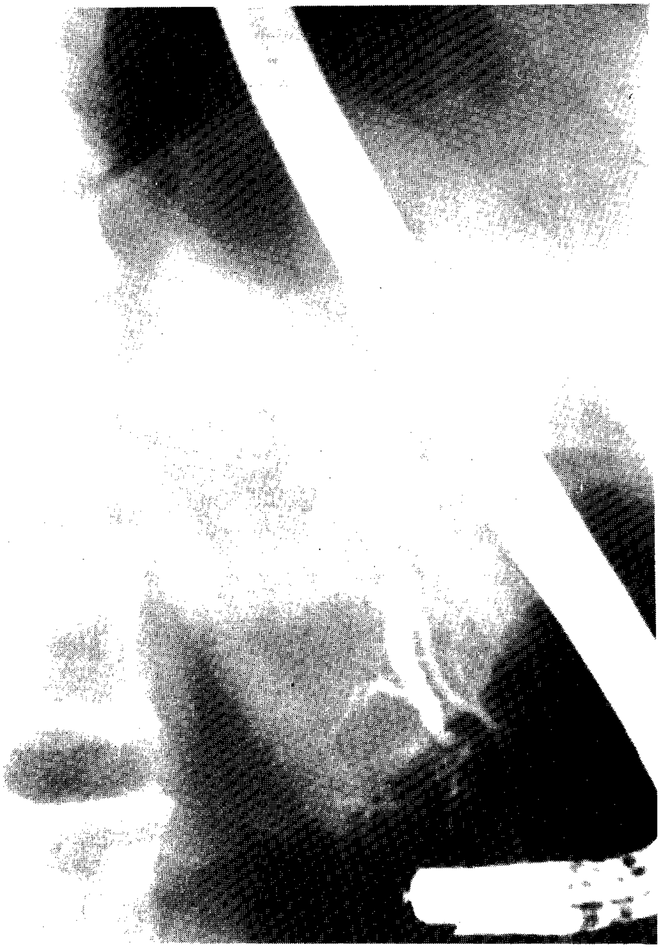

**Figure 4** Pancreatography of Type II.

The distribution of the method of therapy is shown in Table 3. Combined external-internal drainage was performed by percutaneous transgastric drainage of the pseudocyst with side-holes of the catheter in the stomach. However, no initial decrease in pseudocyst size was observed upon keeping the catheter closed for external drainage. It was thereafter opened, and started to work. Therefore the case is here classified in the group of external drainage.

No haemorrhagic or septic complications occurred. One patient developed pancreatocutaneous fistula after percutaneous external drainage. The fistula closed spontaneously but recurrent pseudocyst soon followed. Three further pseudocyst recurrences took place. Total recurrence incidence was 27%.

Table 4 shows the success of therapy according to ERCP classification. Two cases with Type II patho-anatomy were managed expectantly and resolution occurred spontaneously in both cases in 8 and 12 weeks, respectively. All the recurrences occurred in external drainage in Type III pseudocysts. One of these recurrences was

**Table 3** Methods of primary therapy of pancreatic pseudocysts.

| | |
|---|---|
| Expectant | 2 (13%) |
| External drainage | 7 (47%) |
| Internal drainage | 2 (13%) |
| gastric | 1 |
| duodenal | — |
| jejunal | 1 |
| Combined internal-external | 1 (7%) |
| Pancreatic resection | 3 (20%) |

**Table 4** Pseudocyst recurrences according to therapy and ERCP classification.

| *Type* | *Expectant* | | *External drainage* | | *Internal drainage* | | *Resection* | |
|---|---|---|---|---|---|---|---|---|
| | *n* | *Recurrences* | *n* | *Recurrences* | *n* | *Recurrences* | *n* | *Recurrences* |
| I | — | — | 3 | 0 | — | — | — | — |
| II | 2 | 0 | — | — | 1 | 0 | 1 | 0 |
| III | — | — | 5 | 4 | 1 | 0 | 2 | 0 |

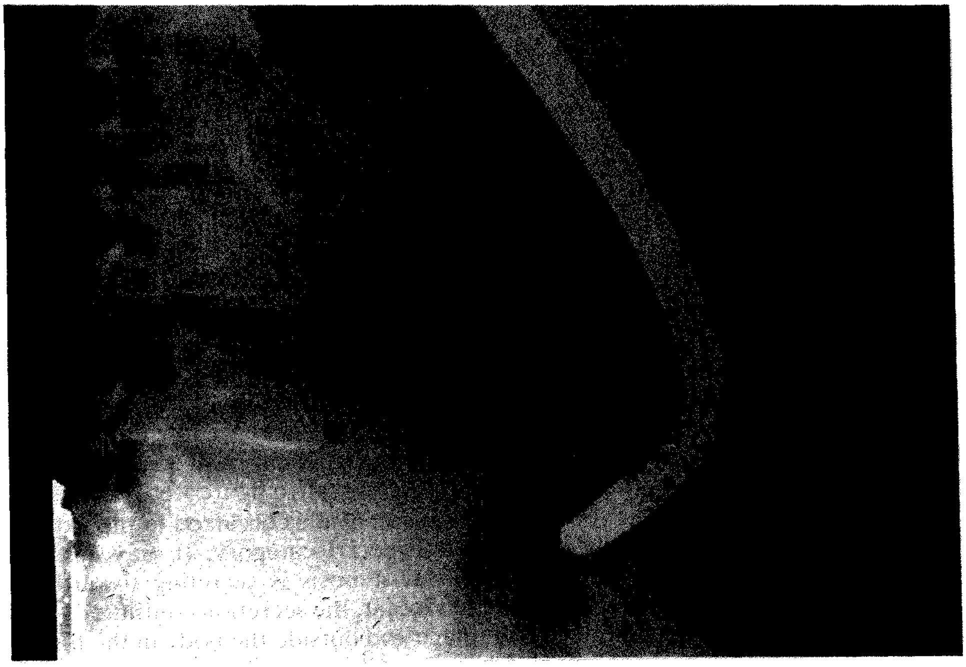

**Figure 5** Pancreatography of Type IIIA.

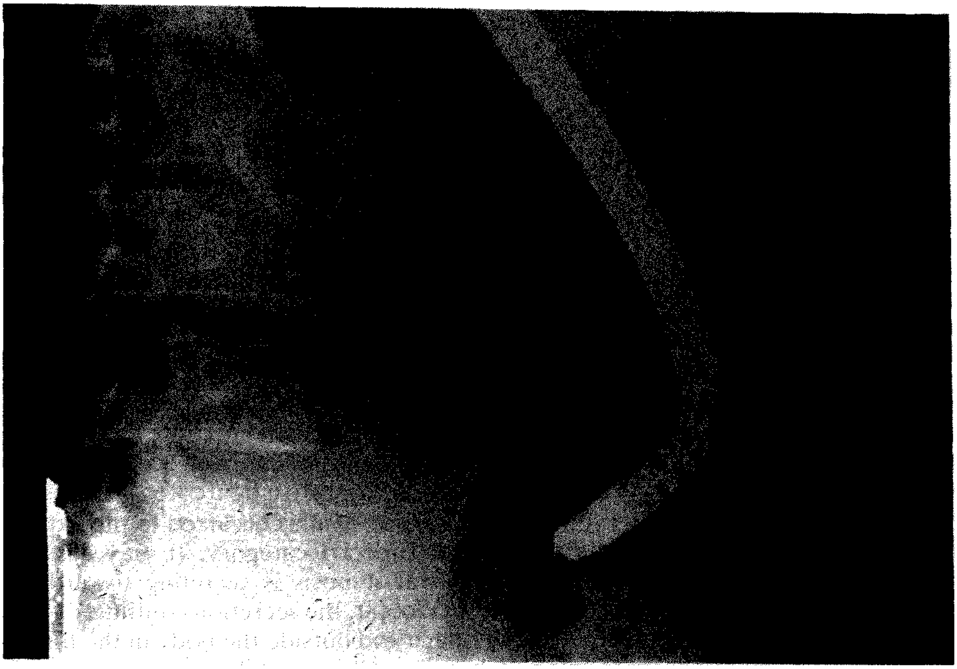

**Figure 6** Pancreatography of Type IIIB.

treated with left pancreatic resection and another with pseudocystojejunostomy, both successfully. The remaining two recurrences are at present under further estimation for treatment.

In the 12 cholangiograms gall-bladder stones were found once. In another case an "open" papillary orifice was seen in duodenoscopy, giving suggestion of a "passed stone"; in biliary radiography no further abnormalities were found.

ERCP-related complications such as septicaemia, acute - phase pancreatitis, cholangitis etc. were never found.

## DISCUSSION

The total number of pancreatic pseudocysts treated in our hospital in the study period was 19; ERCP was attempted on 18 of these. The success rates of pancreatography and cholangiography in patients with pancreatic pseudocysts have been reported to be 80–90%[5,7] and around 70%[6], respectively. The present figures (83%, 67%) can be considered comparable. The lower figure of bile-duct cannulation success is possibly due to lower enthusiasm for bile-tract pathology in cases with alcoholic aetiology of the disease.

Since Amman's first report[9] of a fatal ERCP complication in a patient with pancreatic pseudocyst, caution has been recommended in performing retrograde pancreatograms in this situation. However, in a recent series of 83 ERCPs for pseudocysts, transient pyrexia was the only complication[5]. In another series a 4%

sepsis frequency was noted (two patients)[7]; the sepsis occurred only if there was a delay in pseudocyst therapy. That 4% is, however, only slightly more than the percentage reported in all ERCP examinations[10]. We had no ERCP complications independent of the delay in pseudocyst therapy in half of the cases. Thus, ERCP can be considered a safe procedure in patients with pseudocysts. The need for immediate therapeutic intervention upon the pseudocyst after ERCP[7] may be held to be questionable[5].

ERCP has been considered important in pseudocyst patients for the investigation of the biliary tract[3]. Biliary pathology has been reported in over half of these cases[7]. The smaller frequency (17%) of biliary pathology here may be due to the overwhelming preponderance of alcohol aetiology.

After the introduction of percutaneous needle aspirations[11,12] and percutaneous external drainage[13] of pseudocysts we became enthusiasts about this kind of therapy, which is minimally burdensome to the patient. The results in these reports were surprisingly good in the light of the 24–36% pseudocyst recurrence rate after surgical external drainage [14–16]. More recently, less encouraging results have been published, with a recurrence rate of up to 47%[8,17]. Here, the respective figure was 50%.

All the present recurrences (one with preceding fistula) occurred in the external drainage group. Moreover, all fell into the Type III category. It may easily be imagined that the pancreas behind the ductal stenosis is secreting towards the pseudocyst. If a canal is opened from the pseudocyst, the secretion continues — into the intestinal canal in cases of internal drainage and outside the body in the form of pancreatic fistula in the cases of external drainage. Closure of the internal or external fistula is then followed by recurrent pseudocyst without recurrence after external drainage. This pseudocyst was very caudal, rather a juxtasplenic pseudocyst with stenosis just at the orifice of the pseudocyst. Thus, the pseudocyst hardly collected any true pancreatic secretes, and could also have been classified in the Type IA category.

Communication of the pseudocyst to the main pancreatic duct has been earlier proposed to be the cause of failure of external pseudocyst drainage[18], although the condition has been considered rare[13,18]. We found clear ductal communication in eight out of 15 cases (53%; Types II and IIIA). Moreover, in Type IIIB communication is also very possible.

We found only anecdotal comments on the importance of ductal strictures in the planning of surgical operations for pseudocysts[5,6]. However, it is standard practice to perform ductography to reveal ductectasiae and ductal strictures when treating chronic pancreatitis operatively[19]. Therefore, it is equally logical to consider pancreatography important in evaluating the method of treatment. This may be performed percutaneously through a pseudocyst puncture or by retrograde endoscopically. We prefer the latter method because it also provides the possibility of examining the biliary tract and shows pancreatic ductal anatomy in the case of non-communicating pseudocysts.

Four of the pseudocysts were 4 cm or less in diameter. Two resolved spontaneously (both Type II) and two were successfully drained externally (Type IB). Small pseudocysts have been said to tend to resolve spontaneously[2]. It could also be claimed that, in cases without clear communication to the main duct (Type I), or with ductal communication but without ductal stenosis (Type II), large pseudocysts are rare, and part of them (Type II) tends to resolve spontaneously by drainage through the pancreatic duct into the duodenum. Here, the pseudocyst size in Types I and II

was 4.5 cm on average, as compared to 9.5 cm in Type III.

In acute pseudocysts the immature cyst wall prevents secure internal drainage[16,20]. Percutaneous external drainage may be performed in cases with rapid enlargement or infection. ERCP, however, may again be called for in cases with suspected biliary pathology. In the three acute pseudocysts studied here, ERCPs visualized the gallstones once.

In conclusion, chronic pseudocysts as well as acute pseudocysts after 6 weeks' follow-up, and without marks of spontaneous resolution, can be safely examined by ERCP. We suppose that in Type I pseudocysts external drainage (percutaneous) should suffice as treatment. In Type II pseudocysts, spontaneous resolution may occur and expectant management is recommended for 12 weeks. Cases with still-persisting pseudocyst as well as those falling in the Type III category should be treated with internal drainage to the stomach (possibly endoscopically or percutaneously)[21–23], duodenum or jejunum, depending on the location. In Type III pseudocysts, caudal resection right of the ductal stenosis is recommended in cases with technical possibilities because closure of the pseudocysto-enterostomy might be followed by a recurrent pseudocyst. If the surgeon still remains aware ot the possibility of multilocated and multiple pseudocysts, a very small recurrence rate may be expected.
